# Predicting fatigue using countermovement jump force-time signatures: PCA can distinguish neuromuscular versus metabolic fatigue

**DOI:** 10.1371/journal.pone.0219295

**Published:** 2019-07-10

**Authors:** Paul Pao-Yen Wu, Nicholas Sterkenburg, Kirsten Everett, Dale W. Chapman, Nicole White, Kerrie Mengersen

**Affiliations:** 1 Australian Research Council Centre of Excellence for Mathematical and Statistical Frontiers, School of Mathematical Sciences, Queensland University of Technology, Brisbane, Queensland, Australia; 2 Strength and Conditioning, Australian Institute of Sport, Bruce, A.C.T., Australia; 3 Research Institute for Sport and Exercise, University of Canberra, Bruce, A.C.T., Australia; 4 Physiology, Australian Institute of Sport, Bruce, A.C.T., Australia; Universita degli Studi di Roma ‘Foro Italico’, ITALY

## Abstract

**Purpose:**

This study investigated the relationship between the ground reaction force-time profile of a countermovement jump (CMJ) and fatigue, specifically focusing on predicting the onset of neuromuscular versus metabolic fatigue using the CMJ.

**Method:**

Ten recreational athletes performed 5 CMJs at time points prior to, immediately following, and at 0.5, 1, 3, 6, 24 and 48 h after training, which comprised repeated sprint sessions of low, moderate, or high workloads. Features of the concentric portion of the CMJ force-time signature at the measurement time points were analysed using Principal Components Analysis (PCA) and functional PCA (*f*PCA) to better understand fatigue onset given training workload. In addition, Linear Mixed Effects (LME) models were developed to predict the onset of fatigue.

**Results:**

The first two Principal Components (PCs) using PCA explained 68% of the variation in CMJ features, capturing variation between athletes through weighted combinations of force, concentric time and power. The next two PCs explained 9.9% of the variation and revealed fatigue effects between 6 to 48 h after training for PC3, and contrasting neuromuscular and metabolic fatigue effects in PC4. *f*PCA supported these findings and further revealed contrasts between metabolic and neuromuscular fatigue effects in the first and second half of the force-time curve in PC3, and a double peak effect in PC4. Subsequently, CMJ measurements up to 0.5 h after training were used to predict relative peak CMJ force, with mean squared errors of 0.013 and 0.015 at 6 and 48 h corresponding to metabolic and neuromuscular fatigue.

**Conclusion:**

The CMJ was found to provide a strong predictor of neuromuscular and metabolic fatigue, after accounting for force, concentric time and power. This method can be used to assist coaches to individualise future training based on CMJ response to the immediate session.

## Introduction

Managing the fatigue response to training is critical to maximise athlete adaptation, whilst concurrently minimising potential injury and avoiding overreaching. It is therefore important for coaches and sports scientists alike to accurately monitor and predict the fatigue status of an athlete to specific training programs. We focus here on fatigue in terms of a decline in the ability of muscle group(s) to generate force [1]. The overall fatigue status of an athlete at any point in a training cycle is multi-faceted, reflecting such aspects as the point in the training and competition cycle, nutritional status, use of recovery techniques and general life stressors [2].

Training programs can be optimised by manipulating training variables within the theoretical constraints of the super-compensation model which requires anticipative management of fatigue to ensure full or partial recovery in time for the next training session [3]. Thus, there is a need to monitor both short term fatigue, typically metabolic in origin, and more prolonged, neuromuscular fatigue. Metabolic fatigue is described as a decrement in muscle force generating capacity as a response to physical exercise that has outstripped the rate of ATP1 replacement. Its effects begin to diminish after a period of five minutes and is generally thought to have dissipated after 3 h [1]. This contrasts with neuromuscular fatigue, which is defined as a prolonged decrease in the muscle’s ability to generate a force or power output after a period of recovery. Neuromuscular fatigue can be present for upwards of 48 h, and can be identified as a compound system with both central and peripheral origins [4].

Practitioners often use the Countermovement Jump (CMJ) test to monitor athlete fatigue, or equivalently recovery status, in terms of neuromuscular and/or metabolic fatigue [5]. In multiple studies, the CMJ is used to characterise fatigue in functional lower body dynamic performance following: (i) acute training interventions [6], or (ii) as a longitudinal monitoring tool [7, 8]. Existing research using dynamic performance tests such as the CMJ are generally focused on discrete time dependent variables [9] derived from the ground reaction force (such as maximal or peak concentric force, power or velocity). They do not investigate variation between athletes in relation to fatigue recovery over time and their effects on individualised, predictive fatigue management. Typically, these studies have been based on mean and peak force values using standard inferential statistical techniques such as ANCOVA [3, 10]. A limitation of these approaches, is that these discrete variables do not explicitly account for the multi-dimensional nature of the data, nor the temporal elements associated with different forms of fatigue. Some authors have more recently sought to account for the time dimensionality of the ground reaction force profile via normalisation techniques [11] or via alternative statistical approaches such as spatial parametric mapping [12].

A more widely known approach is the use of Principal Components Analysis (PCA) which can provide a method for explaining variation in data through transformation of many, possibly correlated, factors into a smaller set of uncorrelated components [13]. Thus, a PCA of force-time curve features across training regimes and recovery times can help identify complex patterns in athlete fatigue status and expressing similarities and differences within the data set [14]. Furthermore this technique reduces the dimensionality of the data, which enhances visualisation and interpretation. This approach, however, can be limited in terms of the number of features that can be studied as PCA returns an incomplete result when the dimensionality exceeds the number of samples [13]. As the dimensionality is the product of the number of features by the number of time points, this can occur quite easily for small datasets. Functional PCA (*f*PCA) can help to address this challenge by approximating the force-time curve as a function. In addition, it can provide insight into the characteristics of the force-time curve itself and their relationship to fatigue [15].

In recent times, more comprehensive features based analysis of the CMJ have been reported in relation to their impact on neuromuscular and other forms of fatigue [16]. However, further investigation of these promising insights has been hampered by the lack of a dataset of sufficiently high resolution in time to help discriminate between neuromuscular versus metabolic fatigue.

In this paper, we analyse a new high resolution dataset to study and model the relationship between training workload and onset of short term metabolic fatigue versus long term neuromuscular fatigue. We study CMJ performance of ten recreational athletes at multiple time points from pre- to 48 h post-training in response to different training loads, namely low, moderate, and high volume repeated sprint training workloads. We apply PCA, *f*PCA and regression analysis, focusing on the concentric phase of the ground reaction force-time profile recorded at each time point pre- and post-training. This approach presents a novel opportunity to classify the underlying effects and dynamics of processes associated with fatigue, and presents an opportunity to use CMJ force-time data to monitor and predict fatigue and recovery time. Such tools and outcomes have the potential to support coaches and sport scientists to proactively manage athlete training and fatigue using non-intrusive monitoring based on the CMJ.

## Methods

### Design

This study used a randomised cross-over design with repeated measures over time. Each participant was requested to complete three randomised repeated sprint running sessions of low, moderate, or high workload, separated by a minimum of 96 h. Participants performed a single set of 5 CMJs before each session, immediately after each session (0), and at 0.5, 1, 3, 6, 24 and 48 h, after each session.

### Subjects

Ten recreationally trained athletes (5 male and 5 female) were recruited to participate during the off-season, with physical characteristics of age: 23.5 y ± 1.5 y (mean ± standard deviation); height: 1.71 m ± 0.1 m; and mass: 71.0 kg ± 12.4 kg. Qualitatively, participants’ level of familiarity with jumping movements was variable; however all participants were defined as non-habituated jumpers as they did not participate in jumping dominated sports but remained physically active. Variability in jump technique was not explicitly studied here; however, its impact on performance across athletes is captured via linear mixed effects modelling (Section Statistical Analysis). All procedures were approved by the University of Canberra ethics committee and written informed consent was obtained from all participants prior to commencing data collection.

### Repeated sprint protocol

Participants randomly completed a low (2 sets), moderate (4 sets), and high volume (6 sets) repeated sprint protocol. Each protocol consisted of multiple sets of 10 x 20 m maximal sprints on a 20 s cycle, with a 180 s inter-set rest. The protocol is similar to other high intensity repeated sprint protocols that generate fatigue [17, 18]. Prior to beginning the first set of any training session each participant completed a standardised warm-up. Participants were instructed to perform each sprint at a maximal intensity and strong verbal encouragement was provided. Each sprint repetition was timed using a single beam infra-red light gate system (Fusion Smartspeed Pro, Summer Park, QLD, Australia) and all repeat sprint training was conducted on an indoor synthetic running track.

### Countermovement jump (CMJ)

At each time point of interest participants performed a single set of 5 CMJ trials with minimal (<20 s) passive recovery between each effort. Each CMJ was performed on a portable force plate sampling at 600 Hz (400 Series Force Plate; Fitness Technology, Adelaide, Australia) with a 400 g aluminium bar held across their shoulders to control for arm swing. Ballistic measurement software (Fitness Technology, Adelaide, Australia) was used to operate the force plate and to record the force-time signal which was then exported for processing and extraction using a custom algorithm developed in the R statistical and data analysis environment [19]. Participants were required to stand in an upright position, with their feet approximately shoulder width apart, perform a rapid self-selected depth counter movement followed by an explosive vertical movement with the instruction to jump as high as possible, whilst keeping the bar firmly against their shoulders. Prior to testing at each test period, each participant completed a standardised warm-up of dynamic movements and a series of submaximal CMJs.

Using the ground reaction force-time data from each CMJ performed, data pertaining only to the concentric phase of the jump was extracted. The concentric phase of the CMJ was defined as “the portion of the jump squat before take-off in which the change in displacement is positive” [20]. This typically corresponded to the portion of the force-time curve from where force output was approximately 10% below standing bodyweight to just before flight phase (force = 0). Although there are many time discrete variables that can be used to characterise CMJ performance [9, 21] this investigation has used the following variables due to the mixed population recruited; peak force relative to bodyweight (_rel_PeakF), peak power relative to bodyweight (_rel_PeakP), time to complete the concentric phase and the time from the beginning of the concentric phase to peak force [22].

The analysis obtained using these four features was compared against an analysis using the concentric portion of the force-time curve. Although more features or a greater portion of the curve could be used, both increase dimensionality substantially as jumps are repeated at multiple time points before and after training. Given the small size of the data [10 recreational athletes), the ability to correctly attribute effects in the model is a significant issue [23]. Therefore, we focused on concentric features and curve in this initial study as they have been shown to be related to force development ability and fatigue [20, 24]. However, the methods can be extended in an obvious manner to include more features and more of the curve with a larger dataset.

### Statistical analysis

The statistical analysis is divided into two sections relating to: (i) identification of metabolic and neuromuscular fatigue using PCA and *f*PCA, and (ii) prediction of fatigue status through mixed effects modelling. All analyses were completed using the R statistical programming environment using the stats, nlme and fda packages [15, 19, 25] for PCA, Linear Mixed Effects (LME) and *f*PCA models, respectively.

#### PCA and fPCA

To assess the impact of different repeated sprint protocols on changes in CMJ performance, the four CMJ features (_rel_PeakF, _rel_PeakP, concentric jump time, concentric time to peak force) were stratified by eight time points creating 32 variables for each participant. Each variable was centred and scaled for subsequent analysis. The PCA was performed with the *prcomp* function from the *stats* package [19], using all 32 variables. The *f*PCA was performed using penalised smoothing to fit a series of b-spline basis functions to the force-time curve for each sample and each time point [15].

Using PCA, the Principal Components (PCs, or eigenvectors) and the corresponding proportions of variance explained (eigenvalues) were computed for each method. *f*PCA also produces PCs but the PCs are eigenfunctions, where each element of the PC vector is a function that maps to the force-time curve. For each PC, the relative importance of the original variables and their inter-relationships were inferred from coefficients (loadings) of the eigenvector or eigenfunction. Additionally, the PCA and *f*PCA scores were used to obtain clusters of similar samples or similar fatiguing effects. Variants of the overall PCA and *f*PCA analyses based on subsets of the data were applied for predictive modelling as described below.

#### Linear mixed effects models and prediction

The prediction of athlete fatigue status following training using the CMJ requires the selection of a force-time curve metric useful for sports performance. The _rel_PeakF variable was selected as it has been reported to provide an accurate account of fatigue experienced by an individual compared to other variables [3]. Importantly, any decrement in muscle/joint force output, as measured by _rel_PeakF, compared to a non-fatigued state is a classical description of fatigue regardless of origin or causation [26, 27]. Specifically, we were interested in _rel_PeakF at 6 h and 48 h post training as they correspond to typical rest periods for athletes completing two training sessions during a day and rest over a weekend, respectively.

The PCA and *f*PCA PCs computed from CMJ measurements at some or all time points pre- and post-training were used as predictors of fatigue via _rel_PeakF. Two baseline models were created using all available data (i.e. all time points) to compute PC1 and PC2 using *f*PCA and thus were the benchmark for _rel_PeakF at 6 and 48 h test intervals. These were compared against 6 and 48 h baseline models computed using PCA. The performance of the better of these two models were compared against PCA and *f*PCA based practical models for _rel_PeakF at 6 and 48 h where PC1 and PC2 were computed using only CMJ measurements pre-, post- and 0.5 h after training. All models included an athlete random effect as summarised in Table 1.

**Table 1:** Summary of predictive models.

RESPONSE	MODEL NAME	MODEL #	EXPLANATORY VARIABLES
**METABOLIC FATIGUE _REL_PEAKF AT 6 H**	6 h fPCA Baseline	1	fPCA PC1 and PC2 (using full dataset), athlete random effect
	6 h PCA Baseline	2	PCA PC1 and PC2 (using full dataset), athlete random effect
	6 h fPCA Practical	3	fPCA PC1 and PC2 (using data up to 0.5 h after training), athlete random effect
	6 h PCA Practical	4	PCA PC1 and PC2 (using data up to 0.5 h after training), athlete random effect
**NEUROMUSCULAR FATIGUE _REL_PEAKF AT 48 H**	48 h fPCA Baseline	5	fPCA PC1 and PC2 (using full dataset), athlete random effect
	48 h PCA Baseline	6	PCA PC1 and PC2 (using full dataset), athlete random effect
	48 h fPCA Practical	7	fPCA PC1 and PC2 (using data up to 0.5 h after training), athlete random effect
	48 h PCA Practical	8	PCA PC1 and PC2 (using data up to 0.5 h after training), athlete random effect

The *nlme* R package [25] was used to build LME models using the maximum likelihood algorithm. Here, variations between athletes were modelled using a random intercept. This way the model can account for the repeated measures nature of the experimental design where there are correlations between measurements of the same individual. Additionally, it captures innate differences in _rel_PeakF levels between athletes.

To assess the validity of the models, leave one out cross validation was employed as there were a limited number of participants (*n* = 10). Mean Squared Error (MSE) in the predicted _rel_PeakF was used to assess predictive performance and the coefficient of determination *R*^2^ was used to assess model fit. Additionally, each of the prediction models were compared against their respective baseline models (Models 1 and 2) using the Likelihood Ratio Test (LRT). The LRT compares the fit of one model against another along with whether the difference is significant [28]. The greater the likelihood ratio, the more pronounced this difference.

## Results

### PCA results

**Fig 1:**
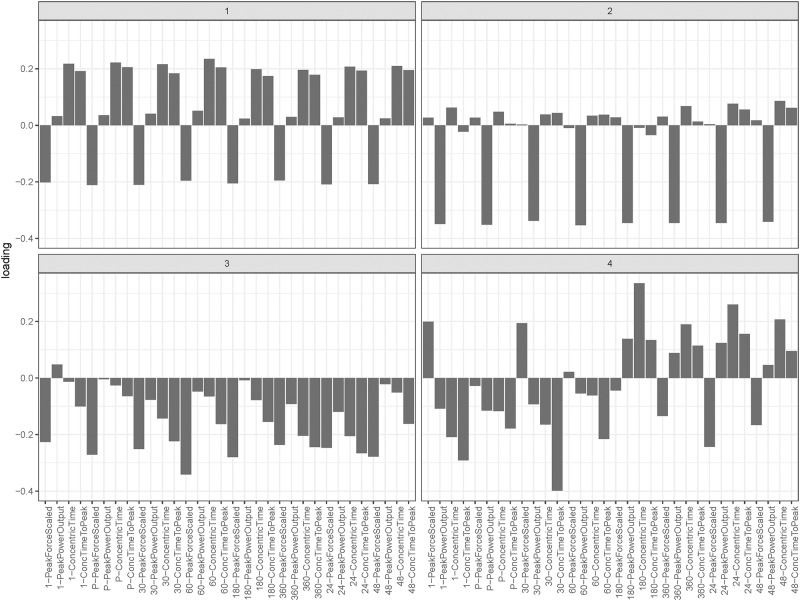
Variable loadings for principal component 1 (PC1) to PC4, showing relative peak force, peak power output, concentric time and concentric time to peak force at each time point. PC1 and PC 2 reflect averaged effects, whereas PC3 shows greater weighting of 6 h to 24 h metrics and PC4 identifies a clear contrast between pre- to 1 h post-training, and 3 h to 48 h post-training effects.

Using the full dataset, which included measurements pre-, post- and up to 48 h after training, the first two PCs obtained through PCA explained 68.7% of the variation in the data combined, and PC3 and 4 explained an additional 9.9%. Together, the first 10 PCs described 93.1% of the variation in the dataset. The loadings of the stratified variables for the first two PCs are illustrated in Fig 1. The loadings for PC1 show a contrast between average force and average time metrics whilst those for PC2 indicate that this PC represents average power. In contrast, PC3 represents a weighted average of peak force, peak power and concurrent time and time to peak force with stronger weighting for the 6 to 24 h period. PC4 demonstrates a clear contrast between loadings pre- to 1 h post training against loadings for 6 h to 48 h.

**Fig 2:**
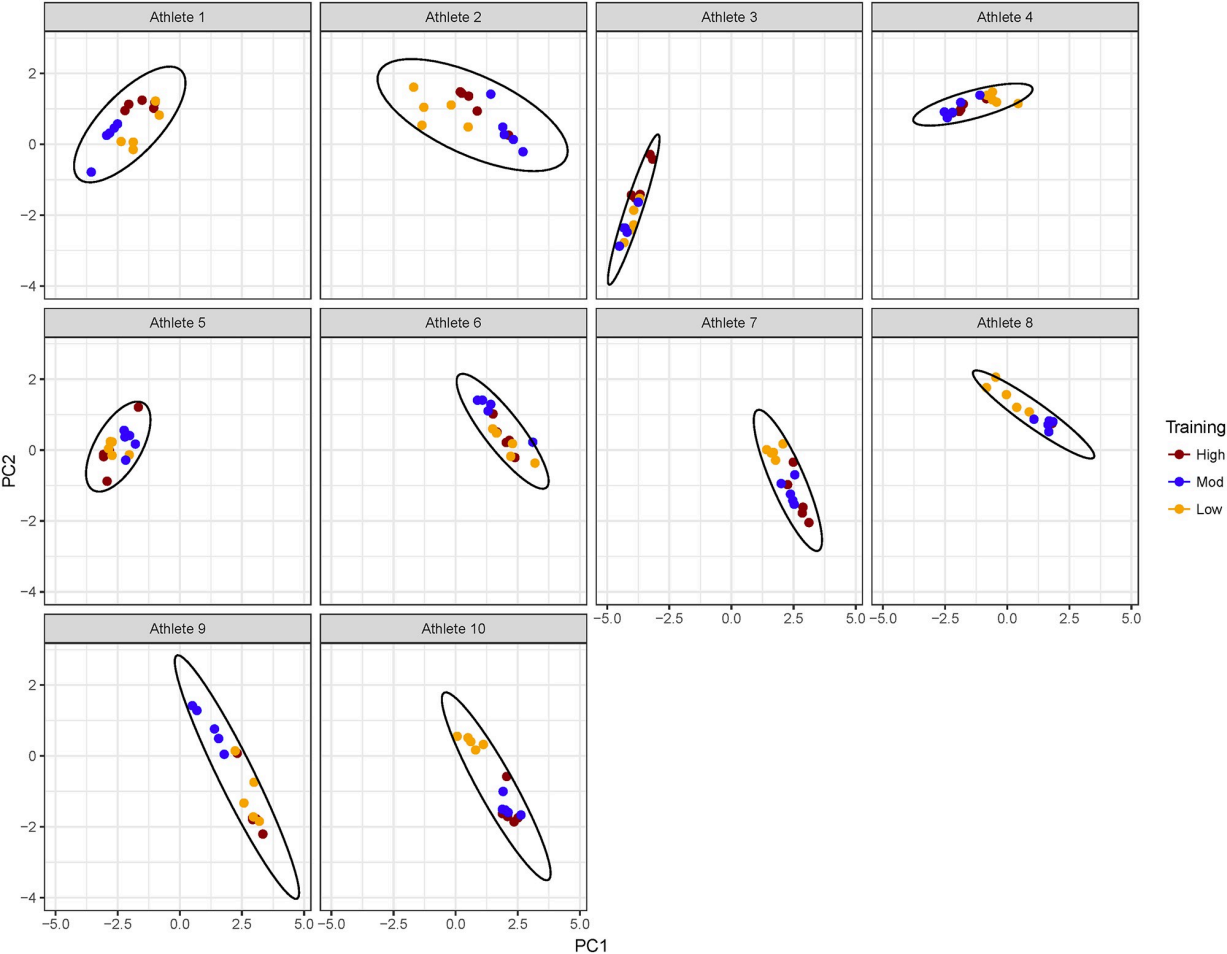
CMJ _rel_PeakF results plotted against PC1 and PC2, illustrating the grouping of individual athlete results and intra-athlete spread of results according to training session workload. CMJ performance tends to cluster by athlete, and within each athlete, there is some separation by training workload.

**Fig 3:**
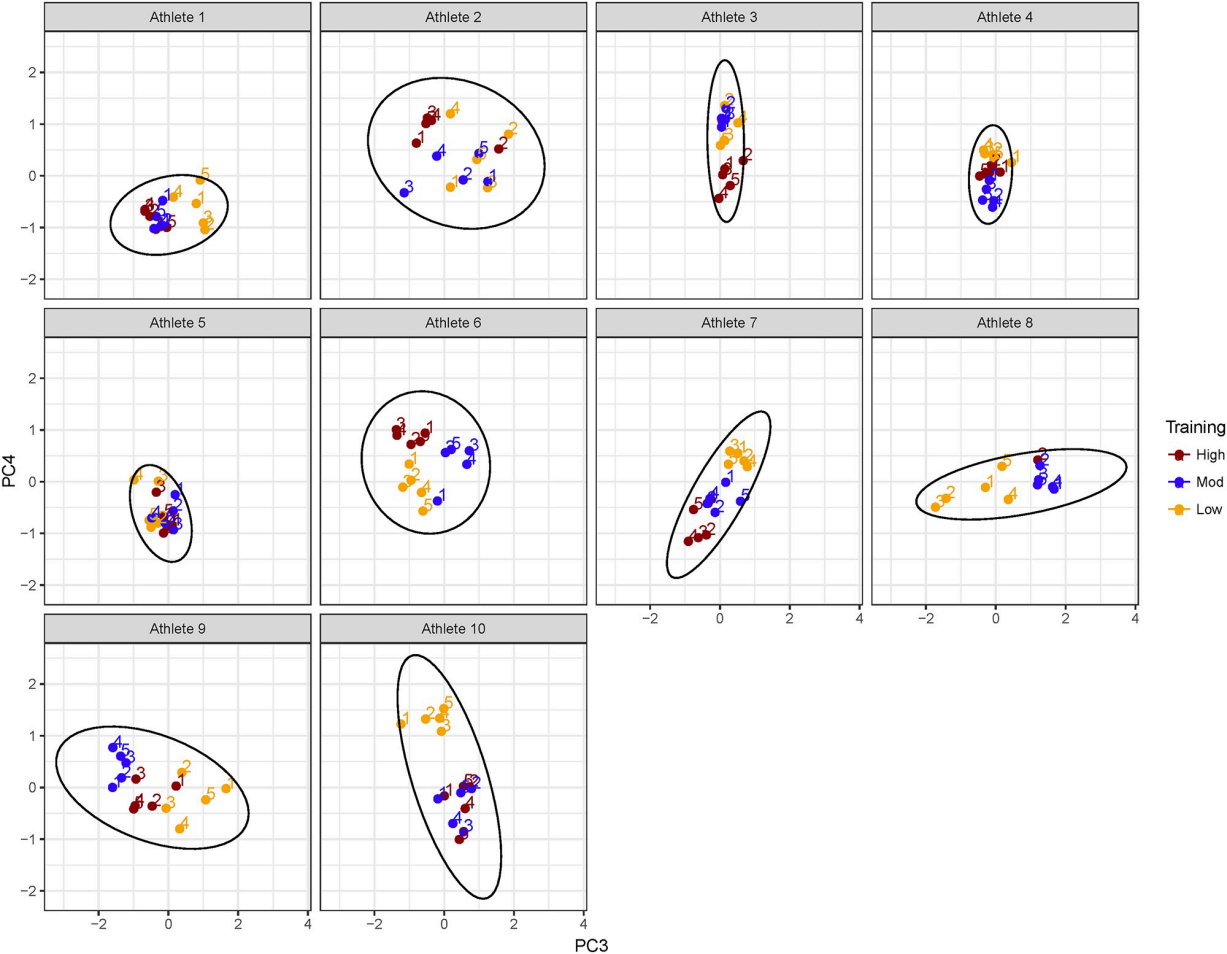
CMJ _rel_PeakF results plotted against PC3 and PC4, illustrating the grouping of individual athlete results showing greater clarity in groupings by training workload for each athlete.

The data for each athlete and each training workload are plotted against PC1 and PC2 in Fig 2 and against PC3 and PC4 in Fig 3. Notably, these analyses revealed that data were clustered most strongly by athlete when plotted against PC1 and PC2 as shown in Fig 2, and then clustered by training workload within each athlete cluster. Clustering of training workload became more apparent when plotted against PC3 and PC4 in Fig 3 with greater separation of high, moderate and low training workloads.

### *f*PCA results

**Fig 4:**
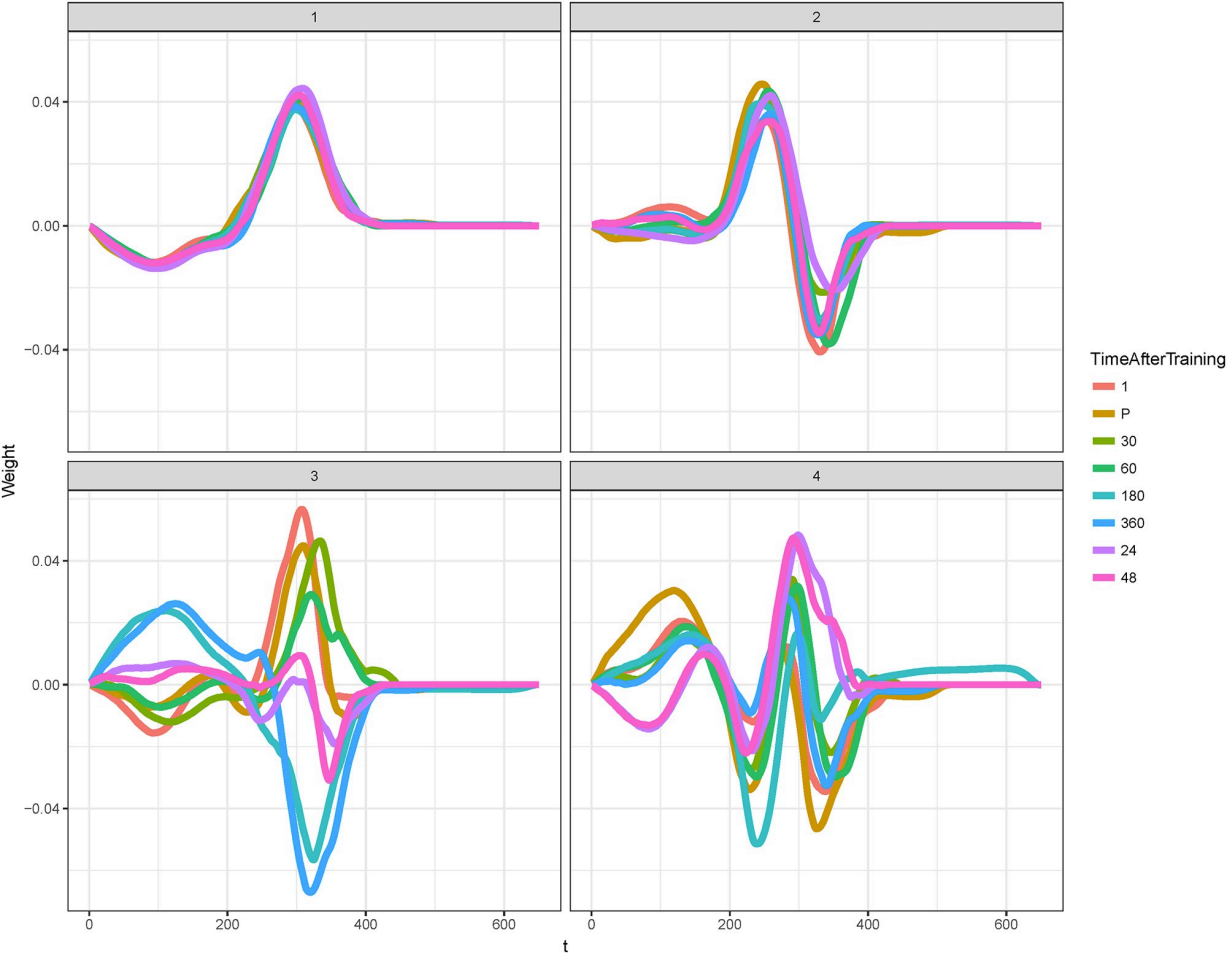
Variable loadings for PC1 to PC4 where each curve represents a stratified time point, the *x* axis the force-curve time and the *y* axis the force-curve weighting. PC1 and 2 represent averaging effects across time points, whereas PC3 and PC4 show contrasts between pre- to 1 h post-training, and 3 h to 48 h post-training. Note also the occurrence of contrasts at the beginning and around the peak of the curve for PC3 and PC4.

The first two PCs obtained with *f*PCA explained 55.8% of the variation whilst PC3 and PC4 explained an additional 8.4%. Similar to PCA results, PC1 is an average of the different time points as is PC2 (Fig 4). However, *f*PCA additionally reveals that PC2 is made up of a contrast between the peak force and the drop to zero. PC3 here shows a contrast between early time points from pre- to 1 h post training and 3 h, 6 h, and 24 and 48 h curves. This contrast occurs at two time intervals on the force-time curve–at the beginning of the concentric phase and around the peak. This contrast is repeated somewhat for PC4 especially at the beginning of the concentric phase, but the latter stage reveals what appears to be contrasting effects associated with two peaks. It was found that a substantial number of CMJs featured two peaks.

**Fig 5:**
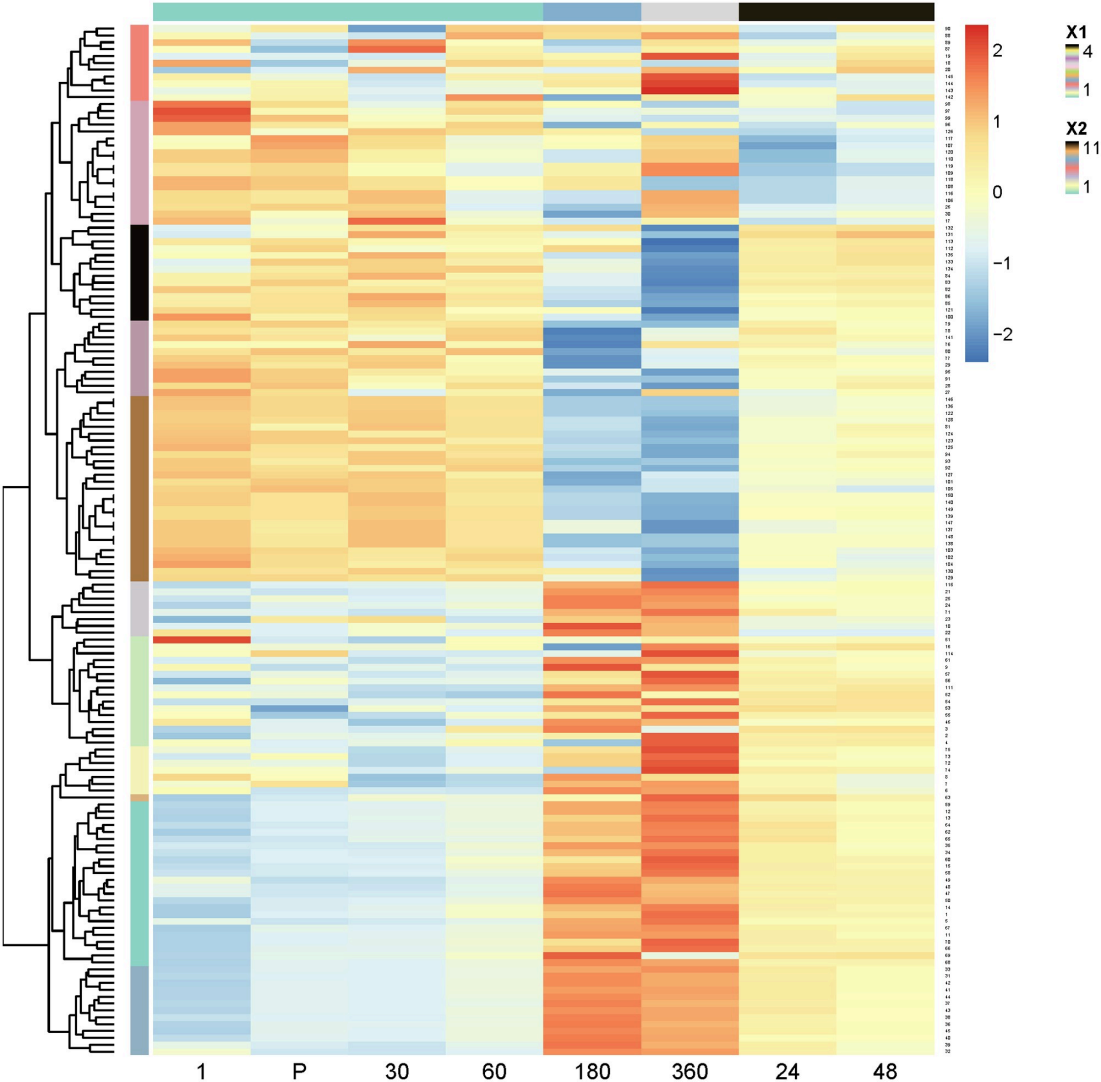
Scores for all 150 sample points (*y* axis) and 8 time points (*x* axis) for PC3. The colour of each cell represents the score value. Clusters of sample points are shown with coloured regions and a tree on the left hand side, and clusters of time points are shown on the top with coloured regions. 1, P, 30, 60 –corresponding to pre-, post-, 0.5 and 1 h after training are clustered together. 3 h and 6 h are in their own clusters, and 24 h and 48 h are clustered together.

The contrast between pre- to 1 h post-training to 3 to 48 h post-training effects as reflected in PC3 and PC4 was reflected in clusters of PC scores for PCs 1 through 4. Hierarchical clustering [29] of *f*PCA scores for each PC revealed that, generally speaking, pre- to 1 h post-training scores were grouped into the same cluster, whilst 3h to 48 h-post training effects tended to be grouped separately (see Fig 5 for PC3). The contrast in scores, especially between larger red versus blue scores (positive versus negative) are generally more prominent within clusters of time points.

### Linear modelling and prediction

Table 2 reports the fit of each model based on cross-validated MSE and the goodness of fit *R*^2^. Perhaps unsurprisingly, the baseline models of _rel_PeakF at 6 h and 48 h demonstrated the best fit (lowest MSE and highest *R*^2^) and predictive accuracy as they used all available data. Both the 6 h and 48 h practical models, which are based on practically obtainable data, vis a vis CMJ measurements taken up to 0.5 h post-training, provided similar cross-validation accuracy to models that used more information. Additionally, *f*PCA and PCA models of the same response and using same time points for PC construction performed similarly in terms of MSE and *R*^2^. Further investigation of the differences between *f*PCA and PCA fitted models using the LRT showed that generally, the fit of *f*PCA based models were not statistically different to their PCA counterpart as reflected in the LRT p-value and minimal differences in predictive MSEs (Table 3). Only the 6 h configuration showed a significant difference in the likelihood ratio but the difference in MSE is small. Significant differences did arise between baseline and practical models in terms of model fit, however, the difference in predictive accuracy (MSE) was small relative to the absolute MSE.

**Table 2:** A list of the linear mixed effects models investigated for the prediction of _rel_PeakF, detailing the covariates in each model (a common covariate in each model was an athlete random effect), mean squared error, cross validation mean squared error and variable selection results.

Model Name	Selected Explanatory Variables	Cross Validation MSE	*R*^2
6 h *f*PCA Baseline	PC1 and PC2 (using *f*PCA on full dataset)	0.009	0.792
6 h PCA Baseline	PC1 and PC2 (using PCA on full dataset)	0.010	0.719
6 h *f*PCA practical	PC1 and PC2 (using *f*PCA on data up to 0.5 h post-training)	0.013	0.640
6h PCA practical	PC1 and PC2 (using PCA on data up to 0.5 h post-training)	0.013	0.639
48 h *f*PCA Baseline	PC1 and PC2 (using *f*PCA on full dataset)	0.015	0.801
48 h PCA Baseline	PC1 and PC2 (using PCA on full dataset)	0.012	0.814
48 h *f*PCA practical	PC1 and PC2 (using *f*PCA on data up to 0.5 h post-training)	0.015	0.714
48 h PCA practical	PC1 and PC2 (using PCA on data up to 0.5 h post-training)	0.015	0.728

**Table 3:** Likelihood Ratio Test results for comparison of the linear mixed effects models used in _rel_PeakF prediction. The ratio describes the increase in goodness of fit (as measured by the log likelihood) for the first model compared to the second model, e.g. the difference in the log likelihood between the 6 h PCA baseline and the 6 h *f*PCA baseline models is 16.32. Here the better model is the one shown to have a statistically significant difference in likelihood.

Response	Model Tested	Likelihood ratio	Difference in MSE	p-value
Metabolic Fatigue	6 h *f*PCA Baseline vs 6 h PCA Baseline	16.32	0.0004	0.003
Neuromuscular Fatigue	48 h *f*PCA Baseline vs 48 h PCA Baseline	2.56	0.0003	0.98
Metabolic Fatigue	6 h PCA practical vs 6 h *f*PCA practical	2.56	0.0009	~1
Neuromuscular Fatigue	48 h PCA practical vs 48 h *f*PCA practical	4.45	0.0009	~1
Metabolic Fatigue	6 h *f*PCA Baseline vs 6 h *f*PCA practical	39.1	0.004	<0.0001
Neuromuscular Fatigue	48 h *f*PCA Baseline vs 48 h *f*PCA practical	27.7	0.0006	<0.0001

## Discussion

The application of PCA to our study of CMJ force-time features at time points before and after training allowed us to identify variables that most strongly contributed to onset of fatigue and recovery following training. We found that PC1 and PC2, which explained the majority of the variation in the data, was dominated by averaging effects across force and time metrics, and of power (Fig 1). This, in combination with the plot of the data on PC1 and PC2 (Fig 2) suggests that differences amongst athletes were the most significant contributor to CMJ variation. However, PC3 and PC4, which explained an additional 9.9% of the variation, exposed contrasts between metabolic fatigue time course indicators for time points pre- to 1 h post-training [1], and neuromuscular fatigue indicators for time points 3 to 48 h post-training [4]. This finding provides evidence to support the use of CMJ as a predictor of neuromuscular and metabolic fatigue.

These findings were validated through *f*PCA evaluation of the concentric portion of the data. PC1 and PC2 again explained the majority of the variation and represented weighted averaging effects over the force-time curve, but additionally revealed a contrast between the beginning of the concentric phase and the peak in PC2. This contrast between phases of the curve and contrasting temporal response between metabolic time points up to 1 h post-training and neuromuscular time points from 3 to 48 h post training were highlighted in PC3. PC4 also demonstrated similar effects but additionally revealed an artefact of jump technique at toe-off which can produce double peaks. We hypothesise that this relates to technical proficiency, coordination and fatigue. When combined with clustering results where scores tended to cluster by time points consistent with metabolic or neuromuscular fatigue but not both, the findings support the use of the CMJ for predicting onset of neuromuscular versus metabolic fatigue.

In addition, coaches and support staff can potentially apply the protocol and PCA and *f*PCA analyses at the beginning of a season and other key points in time to develop team capability maps. These can be used to compare athletes easily and non-invasively based on their metabolic and neuromuscular response to physical activity. This allows coaches to optimise team structure, particularly in sports such as soccer and rugby, to ensure player’s strengths are being adequately utilised. Moreover, it would also serve as a performance monitoring process of an athlete’s response to a given training regime over time. The cautionary point to this approach is that there was noticeable intra-trial fatigue in our participants when executing the proposed protocol (Section Design), i.e. fatigue experienced when performing 5 consecutive CMJs, as evident in the spread of performance results within the athlete and training intensity clusters (Fig 2). However, this dispersion was generally less than the variation due to difference in training intensities and between individual athletes. It would also be reasonable to assume that in a suitable athletic population that has a higher training age, the individual variation in performing 5 consecutive CMJs would reduce thus improving the predictive accuracy of the described approach.

Since PCs can be considered as variables themselves, they can be used in fatigue prediction models based on CMJ measurements at different times before and after training. The advantage of using *f*PCA results is that the entire curve along with any peculiarities such as a double peak can be used to predict fatigue. Using linear mixed effects models we were able to predict the onset of neuromuscular and metabolic fatigue based on _rel_PeakF whilst accounting for large variations in fatigue profile between athletes. Practically, using CMJ measurements up to 0.5 h after training (athletes are likely to still be at the training facility at this time), we were able to predict the _rel_PeakF of the CMJ with a MSE of 0.013 and 0.015 at 6 and 48 h, corresponding to metabolic and neuromuscular fatigue, respectively. While the baseline model was significantly better than the practical model, the practical model was still comparable in cross-validated predictive performance to the baseline models. Therefore, the study suggests that it is feasible to use CMJ measurements collected before and up to 0.5 h post training to help predict the onset of, and hence manage, neuromuscular and metabolic fatigue.

Somewhat surprisingly, the predictive accuracy (i.e. MSE) of models using PCA were virtually identical to those using *f*PCA across baseline and practical models in predicting both metabolic and neuromuscular fatigue. This suggests that for our dataset, the four variables of peak force, peak power, concentric time and time to peak are good predictors of neuromuscular versus metabolic fatigue. However, it is possible that a larger sample size would enable *f*PCA based models, which are of higher dimensionality than discrete PCA based models, to achieve greater predictive accuracy as they can better capture jump and fatigue variability.

## Practical applications

In an application of these findings we propose that the use of the 6 and 48 h predictions can serve very different purposes in the training optimisation context. Athletes in many sports will commonly complete two training sessions each day, and the 6 h prediction model can be used to assess training readiness of an athlete ahead of their second daily session. In contrast, as the 48 h prediction model is more strongly related to the fatigue status of an athlete following a training session or possibly match play, this model can be used to predict fatigue status following a programmed rest day, for example. For coaches and athletes, ensuring maximum training benefit is achieved while avoiding overreaching and injury is of extreme importance. By having an almost real time understanding of an athlete’s response to a training session, as well as a prediction of their fatigue condition prior to subsequent sessions, these detrimental training by-products or training errors can be avoided [30]. Interestingly, the PCA athlete capability map (Fig 2) provided empirical evidence of athlete pacing during some training sessions, as fatigue experienced from moderate training workloads exceeded that experienced from high training workload. Such findings may be useful to coaches in the provision of training sessions. However, pacing effects are beyond the scope of this current work.

Although _rel_PeakF variable has been shown to be an accurate indicator of fatigue [3], there are many other sport-specific variables relevant to fatigue and performance that could be predicted or studied using the developed PCA/*f*PCA approach. Furthermore, PCA/fPCA outcomes could be related to sport-specific performance metrics. For example, Mooney, Cormack et al. (2013) [31] observed that the CMJ flight time to contraction time ratio (FT:CT) was predictive of practical metrics of match intensity (measured with accelerometers) and performance (assessed by coaches) in Australia Rules Football. Relating PCA/fPCA outcomes to sport-specific, practical metrics can assist coaches and athletes to better manage their workloads in view of their impact on in-game performance. As a result, another key outcome of our work is the method itself, as it can be translated by or for practitioners to relate to fatigue and performance indicators directly relevant to their sport.

Despite the suitability of the developed algorithm to the analysis of any CMJ data, the number of participants in this investigation can be considered small in comparison to the number of variables in the analysis. This limits the number of features that can be used for PCA analysis and the degree of complexity in the force-time curve that can be captured due to model identifiability with high dimensionality and small sample size. However, the methods can be applied and extended to model more complex curves such as including eccentric and concentric phases [16, 32, 33] and other features (e.g. velocity) given a larger sample size. Therefore, further study using a broader data set, such as with differing fatiguing stimulus other than repeated sprints and athletes of differing training status (elite versus sub-elite for instance), can help to validate the findings of this study. Our study revealed that the greatest variability in PC1 and 2 was due to differences among athletes, which suggests that jump technique could be a key factor affecting CMJ performance worthy of future research. Additional validation can be provided through cross validation of the results of this investigation with other alternative fatigue characterisation methods such as plasma blood lactate analysis. Such further study can also provide greater insight into the dynamics of neuromuscular and metabolic fatigue onset. Finally, although _rel_PeakF was used as one relevant indicator of CMJ performance and fatigue, future work with larger datasets can additionally investigate techniques such as functional linear mixed effects modelling to model the force-time curve directly using functional data [15].

## Conclusions

In this paper, we related neuromuscular and metabolic fatigue to the first four principal components of a PCA and *f*PCA of the concentric portion of CMJs at time points before and after training. This enabled us to develop athlete capability maps and, with the use of linear mixed effects models, predict the fatigue status of athletes at 6 and 48 h after training sessions of differing workloads based on _rel_PeakF. Such a tool has the potential to support coaches and athletes in managing training workload with respect to fatigue.

## Supporting information

S1 Data Countermovement Jump (CMJ) data used in this study. Ten athletes participated and each participant was requested to complete three randomised repeated sprint running sessions of low, moderate, or high workload, separated by a minimum of 96 h. Participants performed a single set of 5 CMJs before each session, immediately after each session (0), and at 0.5, 1, 3, 6, 24 and 48 h, after each session.
(CSV)

S1 Table Summary statistics for the standard deviation, proportion of variance and cumulative proportion of the principal components (PCs) calculated from the CMJ data. The PCs are a transformation of the data where PC1 describes the most variation in the data, followed by PC2 and so on. In our study, PC1 corresponds to neuromuscular fatigue effects and PC2 to metabolic fatigue effects.
(DOCX)
