# Supplementary material for: Predicting fatigue using countermovement jump force-time signatures: PCA can distinguish neuromuscular versus metabolic fatigue
Source: PLoS One. 2019 Jul 10;14(7):e0219295. doi: 10.1371/journal.pone.0219295 (PMC6619745; doi:10.1371/journal.pone.0219295)
Supplement: S1 Table — The PCs are a transformation of the data where PC1 describes the most variation in the data, followed by PC2 and so on. In our study, PC1 corresponds to neuromuscular fatigue effects and PC2 to metabolic fatigue effects. (DOCX) [file pone.0219295.s002.docx]

# Supplementary Material

S1 Table. *Summary statistics for the standard deviation, proportion of variance and cumulative proportion of the principal components (PCs) calculated from the CMJ data. The PCs are a transformation of the data where PC1 describes the most variation in the data, followed by PC2 and so on. In our study, PC1 corresponds to neuromuscular fatigue effects and PC2 to metabolic fatigue effects.*

| Variables | PC1 | PC2 | PC3 | PC4 | PC5 | PC6 | PC7 | PC8 | PC9 | PC10 | PC11 | PC12 | PC13 |
| --- | --- | --- | --- | --- | --- | --- | --- | --- | --- | --- | --- | --- | --- |
| Standard deviation | 3.847 | 2.680 | 1.377 | 1.122 | 1.103 | 0.966 | 0.904 | 0.791 | 0.739 | 0.712 | 0.558 | 0.504 | 0.478 |
| Proportion of Variance | 0.462 | 0.224 | 0.059 | 0.039 | 0.038 | 0.029 | 0.026 | 0.020 | 0.017 | 0.016 | 0.010 | 0.008 | 0.007 |
| Cumulative Proportion | 0.462 | 0.687 | 0.746 | 0.786 | 0.824 | 0.853 | 0.878 | 0.898 | 0.915 | 0.931 | 0.940 | 0.948 | 0.956 |
